# Comparison of Quality Performance Measures for Patients Receiving In-Person vs Telemedicine Primary Care in a Large Integrated Health System

**DOI:** 10.1001/jamanetworkopen.2022.33267

**Published:** 2022-09-26

**Authors:** Derek J. Baughman, Yalda Jabbarpour, John M. Westfall, Anuradha Jetty, Areeba Zain, Kathryn Baughman, Brian Pollak, Abdul Waheed

**Affiliations:** 1 Robert Graham Center, Policy Studies in Family Medicine and Primary Care, Washington, DC; 2 Family Medicine Residency Program, WellSpan Good Samaritan Hospital, Lebanon, Pennsylvania; 3 WellSpan Online Primary Care, York, Pennsylvania

## Abstract

**Importance:**

Despite its rapid adoption during the COVID-19 pandemic, it is unknown how telemedicine augmentation of in-person office visits has affected quality of patient care.

**Objective:**

To examine whether quality of care among patients exposed to telemedicine differs from patients with only in-person office-based care.

**Design, Setting, and Participants:**

In this retrospective cohort study, standardized quality measures were compared between patients with office-only (in-person) visits vs telemedicine visits from March 1, 2020, to November 30, 2021, across more than 200 outpatient care sites in Pennsylvania and Maryland.

**Exposures:**

Patients completing telemedicine (video) visits.

**Main Outcomes and Measures:**

χ^2^ tests determined statistically significant differences in Health Care Effectiveness Data and Information Set (HEDIS) quality performance measures between office-only and telemedicine-exposed groups. Multivariable logistic regression controlled for sociodemographic factors and comorbidities.

**Results:**

The study included 526 874 patients (409 732 office-only; 117 142 telemedicine exposed) with a comparable distribution of sex (196 285 [49.7%] and 74 878 [63.9%] women), predominance of non-Hispanic (348 127 [85.0%] and 105 408 [90.0%]) and White individuals (334 215 [81.6%] and 100 586 [85.9%]), aged 18 to 65 years (239 938 [58.6%] and 91 100 [77.8%]), with low overall health risk scores (373 176 [91.1%] and 100 076 [85.4%]) and commercial (227 259 [55.5%] and 81 552 [69.6%]) or Medicare or Medicaid (176 671 [43.1%] and 52 513 [44.8%]) insurance. For medication-based measures, patients with office-only visits had better performance, but only 3 of 5 measures had significant differences: patients with cardiovascular disease (CVD) receiving antiplatelets (absolute percentage difference [APD], 6.71%; 95% CI, 5.45%-7.98%; *P* < .001), patients with CVD receiving statins (APD, 1.79%; 95% CI, 0.88%-2.71%; *P* = .001), and avoiding antibiotics for patients with upper respiratory infections (APD, 2.05%; 95% CI, 1.17%-2.96%; *P* < .001); there were insignificant differences for patients with heart failure receiving β-blockers and those with diabetes receiving statins. For all 4 testing-based measures, patients with telemedicine exposure had significantly better performance differences: patients with CVD with lipid panels (APD, 7.04%; 95% CI, 5.95%-8.10%; *P* < .001), patients with diabetes with hemoglobin A_1c_ testing (APD, 5.14%; 95% CI, 4.25%-6.01%; *P* < .001), patients with diabetes with nephropathy testing (APD, 9.28%; 95% CI, 8.22%-10.32%; *P* < .001), and blood pressure control (APD, 3.55%; 95% CI, 3.25%-3.85%; *P* < .001); this was also true for all 7 counseling-based measures: cervical cancer screening (APD, 12.33%; 95% CI, 11.80%-12.85%; *P* < .001), breast cancer screening (APD, 16.90%; 95% CI, 16.07%-17.71%; *P* < .001), colon cancer screening (APD, 8.20%; 95% CI, 7.65%-8.75%; *P* < .001), tobacco counseling and intervention (APD, 12.67%; 95% CI, 11.84%-13.50%; *P* < .001), influenza vaccination (APD, 9.76%; 95% CI, 9.47%-10.05%; *P* < .001), pneumococcal vaccination (APD, 5.41%; 95% CI, 4.85%-6.00%; *P* < .001), and depression screening (APD, 4.85%; 95% CI, 4.66%-5.04%; *P* < .001).

**Conclusions and Relevance:**

In this cohort study of patients with telemedicine exposure, there was a largely favorable association with quality of primary care. This supports telemedicine’s value potential for augmenting care capacity, especially in chronic disease management and preventive care. This study also identifies a need for understanding relationships between the optimal blend of telemedicine and in-office care.

## Key Points

**Question:**

Is there a difference in standardized quality performance measures for primary care patients exposed to telemedicine compared with patients with office-only (in-person) care?

**Findings:**

In this cohort study of 526 874 patients, telemedicine exposure was associated with significantly better performance or no difference in 13 of 16 comparisons, mostly in testing-based and counseling-based quality measures. Patients with office-only visits had modestly better performance in 3 of 5 medication-based quality measures.

**Meaning:**

Findings suggest that telemedicine exposure in primary care poses a low risk for negatively affecting quality performance, highlighting its potential to suitably augment care capacity.

## Teaser

This cohort study examines whether quality of care among primary care patients exposed to telemedicine differs from those with only in-person office-based care.

## Introduction

Telemedicine offers great potential for promoting the quadruple aim in health care delivery: patient experience, clinician experience, cost, and quality.^1^ Studies on patient experience have shown high satisfaction with telemedicine,^2,3,4,5^ citing convenience, higher levels of comfort, and decreased costs.^4^ Studies on clinician experience have shown improved intra-office effectiveness, patient-clinician communication, and improved outcomes (notably in medication adherence).^2,6^ Regarding health care costs, telemedicine has been shown to favorably affect office management costs, revenue (by decreased staffing requirements and extending office hours),^7,8^ and no-show rates.^9,10^ Telemedicine has also shown potential for improving clinical outcomes, especially in managing chronic diseases like diabetes and hypertension,^11,12,13,14,15,16^ reducing diagnostic times, and reducing infection risk.^9,17,18^ Certainly, telemedicine has its limitations, notably in the heterogeneous evidence surrounding its long-term cost-effectiveness,^5,7,8,19^ the known variability in telemedicine’s quality of care,^20^ and its association with follow-up care.^21^

Rapid changes in the health care landscape due to the COVID-19 pandemic facilitated a surge of telemedicine, accounting for substantial portions of total ambulatory visits in multiple countries, including the United States.^22^ Despite widespread use, the quality of this broad increase of telemedicine utilization has not been well studied.^23^ Moreover, the comparison of quality between office and telemedicine care during COVID-19 is largely lacking. Although Medicare’s 1135 waiver has granted authority for broad reimbursement of telemedicine services for all eligible beneficiaries until the pandemic resolves (including the elimination of cost sharing),^24^ flexibility of this waiver may wane without evidence of telemedicine’s value. And as health care leaders have emphasized, there is a need to understand telemedicine’s quality in primary care, especially its efficacy as a cost-effective, patient-centered means of care-delivery following the pandemic.^25^

To study quality performance, industry-standard measures, referred to generally as Healthcare Effectiveness Data and Information Sets (HEDIS), are used by hospitals, health care systems, and government agencies (including the Centers for Medicare & Medicaid Services [CMS]) for national reporting on quality of care.^26^ Such measures endorsed by the National Quality Forum (NQF)^27^ (a large stakeholder consortium of industry leaders from medical associations to insurance companies) have quality clout for researchers. As national claims-based data sets have limited granularity for comparing specific quality performance measures between telemedicine and office-based groups, health system data remain the leading approach for evaluating the outcomes of telemedicine exposure.

An unanswered question remains: given the unprecedented increase in telemedicine use, does exposure to this type of care affect quality? Thus, the objective of this study was to compare the quality of telemedicine and in-office visits with standardized HEDIS performance measures during the COVID-19 telemedicine surge.

## Methods

This retrospective cohort study evaluated the outcomes of telemedicine exposure during the COVID-19 pandemic in a large, integrated health system spanning central Pennsylvania and northern Maryland (WellSpan Health, with 8 hospitals, 20 000 employees, and 2600 clinicians). The cohort was divided by patients (not visits): those with office-only (in-person) visits and those with exposure to telemedicine (either only video visits or a blend of video and office). Deidentified, secondary data were extracted from the electronic medical record (EMR) to compare HEDIS quality performance from March 1, 2020, to November 30, 2021 across more than 200 outpatient care sites between the divided cohort. The study was deemed exempt from full board review by the WellSpan Health institutional review board, and informed consent was waived owing to the use of deidentified data. The reporting followed Strengthening the Reporting of Observational Studies in Epidemiology (STROBE) reporting guideline for cohort studies.

### Measure Selection

Sixteen total HEDIS measures were selected across 5 domains of primary care: cardiovascular care, diabetes, prevention and wellness, behavioral health, and pulmonary care. The NQF Core Quality Measures Collaborative consensus core set for accountable care organizations, patient-centered medical homes, and primary care^28^ was used as a template, but we included additional measures in the prevention domain (influenza and pneumococcal vaccination rates) and cardiovascular domain (antiplatelet therapy, lipid panel, and β-blocker therapy) validated by the CMS within the Comprehensive Primary Care Initiative (CPC and CPC+).^29^ Details regarding quality measure selection are outlined in eTable 2 in the Supplement. When quality measures were vague in describing either the measurement period (eg, seasonal timeframes for influenza vaccination) or types of tests included in performance calculations (eg, variations of fecal DNA tests for colon cancer screening), specification clarity was obtained from the United States Preventive Services Task Force (USPSTF).^30^ When redundancies or conflicting details emerged between organizations (eg, numerator and denominator inclusions, exclusions, and time frames), we adhered to a specification hierarchy: NQF, then USPSTF, then CMS. For ease of interpretation, the quality measures were arranged into 3 categories: medication, testing, and counseling.

### Cohort Design

The purpose of cohort division was to compare patients with and without exposure to telemedicine and to evaluate any differences in quality performance. Using EPIC’s SlicerDicer, a robust EMR-based clinical data mining and analysis tool,^31^ we filtered patients in hierarchical stages to compare the HEDIS-specified performance between patients with only office visits (a filter excluding all patients with telemedicine visits) vs patients with exposure to telemedicine (either having only telemedicine visits or a blend of office and telemedicine). Telemedicine encounters included only video visits (not telephone) to exclude encounters such as medication refills, nursing triage, or billing and result notifications. To ensure demographic comparability, we first evaluated telemedicine exposure in 2 groups (patients with a blend of office and telemedicine vs patients with only telemedicine visits), but then combined the telemedicine groups during statistical analysis. The conceptual approach to hierarchical stages and quality measure data extraction with SlicerDicer is represented schematically in the Figure. HEDIS steward specifications for each performance measure are detailed in eTable 2 in the Supplement.

**Figure.**
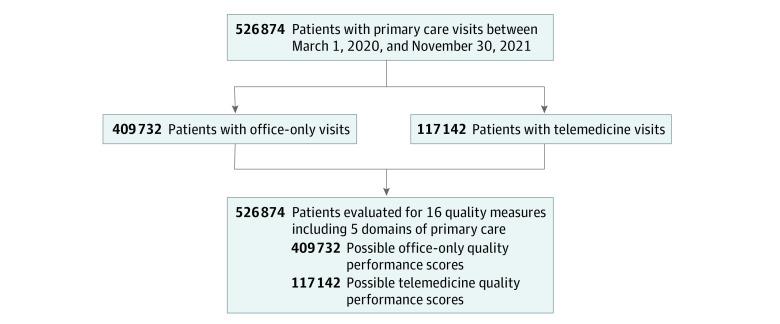
Schema for SlicerDicer Quality Measure Mining and Cohort Divisions This flow diagram represents the data event template for each of the 16 quality of care measures. The data sessions for each of the 16 quality measures were constructed from the specifications of the measure steward. The following hierarchy of filtering would select a patient eligible for a quality performance score: (1) patient had a face-to-face encounter (in-office or telemedicine) with a clinician in the primary care service line within the study timeframe, (2) the patient met unique criteria for the quality measure (patterned build in SlicerDicer with the data filters outlined in eTable 2 in the Supplement), (3) measure steward specified data filters for diagnosis, age, and/or testing were applied. To facilitate the comparison by exposure type, this schema was completed twice to sort patients with telemedicine visits (including those with only telemedicine visits and those with both in-person and telemedicine visits) vs patients with only office visits. This was accomplished by combining an inclusion filter for office encounter with an exclusion filter for telemedicine encounter to obtain the office-only patients and then vice-versa for patients with telemedicine exposure.

### Time Frame Selection, Bias Controls, and Variables

We selected an uninterrupted pandemic-specific time frame^32^ from March 1, 2020, to November 30, 2021, to preserve validity in quality performance reporting during COVID-19 (according to NQF^33^). Similar to recent studies,^34^ we controlled for selection bias with a pre–COVID-19 historical baseline of quality performance (January 18, 2018, to February 29, 2020) to facilitate comparative context to the study timeframe and to provide reassurance of data consistency in representing the population over time (eTable 1 in the Supplement). Sampling bias was further addressed with a regression model to control for sociodemographic factors of telemedicine exposure (eTable 3 in the Supplement), filtering results to include only patients seen in primary care service line, and specific inclusion criteria detailed by the measure steward (eTable 2 in the Supplement).

We identified several variables including: encounter type (office and telemedicine), diagnosis based on measure specification, and clinician type. Possible biases and confounders included COVID-19 pandemic, age, race and ethnicity, sex, and social determinants of health (SDOHs). To control for these, we obtained deidentified demographic data stratified by age, race and ethnicity, sex, and SDOH. Race and ethnicity were self-reported on EMRs; racial categories included American Indian, Alaska Native, Native Hawaiian and other Pacific Islander; Asian; Black or African American; unknown, declined, or not reported; White; and other. Ethnic categories included Hispanic or Latino; not Hispanic or Latino; and other. Patients selected other when they did not identify as the other listed racial and ethnic groups or to indicate being multiracial. We used an overall adult risk score, defined according to the health system as an indicator of overall health risk based on the number of high-risk diagnoses, SDOHs, and health care utilization (including emergency department use and hospital admissions). As discussed in the Supplement of our prior publication,^35^ the SDOH component of this overall health risk score included assessment of social risk needs (depression risk, tobacco and alcohol use, violence exposure and social isolation, food or transportation insecurity, and financial strain), providing a more granular risk profiling compared with other standardized scoring systems.^36,37^

### Statistical Analysis

Absolute percent differences (APDs) between the 16 quality measures were calculated for office and telemedicine encounters. χ^2^ tests were used to test significant differences, with *P* < .01 set as the level of statistical significance. To observe whether age, gender, race and ethnicity, or comorbidities affected results, we used nonaggregated patient level data from SlicerDicer to perform multivariable logistic regression. HEDIS measures with the highest patient volume were selected from different care domains, and separate regression models were completed for the standardized quality outcomes: statin therapy, influenza vaccination, hypertension, and depression screening (eAppendix in the Supplement).

## Results

There were 526 874 patients (409 732 office-only; 117 142 telemedicine exposed) who met inclusion criteria during the study timeframe (Table 1). Demographic analysis by office-only vs telemedicine exposure (blended telemedicine/office and telemedicine-only groups) revealed comparable distributions of representation between the office-only and telemedicine-exposed groups. The majorities of the population were: White (334 215 [81.6%] and 100 586 [85.9%]), non-Hispanic (348 127 [85.0%] and 105 408 [90.0%]), and female (196 285 [49.7%] and 74 878 [63.9%] women). The highest representation of age were adults ages 18-65 (239 938 [58.6%] and 91 100 [77.8%]), and most patients had low risk health scores (373 176 [91.1%] and 100 076 [85.4%]). The single largest group of payers was commercial insurance (227 259 [55.5%] and 81 552 [69.6%]), but Medicare and Medicaid combined represented nearly half of patients (176 671 [43.1%] and 52 513 [44.8%]). A historical baseline vs study time frame comparison between office-only and telemedicine-exposed groups similarly revealed largely comparable numbers of patients between groups and time frames, and there were no overwhelmingly large increases or decreases in quality performance, notably for the office-only group (eTable 1 in the Supplement).

**Table 1.** Population Demographic Data, Overall and by Telemedicine Exposure

Characteristic	Patients, No. (%)			
	Office-only	Telemedicine exposed		Total patients
		Blended office/telemedicine	Telemedicine-only	
No. (%)	409 732 (77.77)	112 199 (21.30)	4943 (0.94)	526 874 (100.0)
Race				
American Indian, Alaska Native, Native Hawaiian and other Pacific Islander	930 (0.23)	320 (0.29)	6 (0.12)	1256 (0.24)
Asian	4482 (1.09)	1020 (0.91)	79 (1.60)	5581 (1.06)
Black or African American	21 673 (5.29)	5800 (5.17)	188 (3.80)	30 361 (5.25)
Unknown, declined, not reported	16 271 (3.97)	2277 (2.03)	182 (3.68)	18 730 (3.55)
White	334 215 (81.57)	96 356 (85.88)	4230 (85.58)	434 801 (82.52)
Other	31 011 (7.57)	7219 (6.43)	267 (5.40)	38 497 (7.31)
Ethnicity				
Not Hispanic or Latino	348 127 (84.96%)	101 064 (90.08)	4344 (87.88)	453 535 (86.08)
Hispanic or Latino	34 588 (8.44%)	7946 (7.08)	291 (5.89)	42 825 (8.13)
Other	27 017 (6.59%)	3189 (2.84)	308 (6.23)	30 514 (5.79)
Legal sex				
Female	196 283 (47.91)	72 226 (64.37)	2652 (53.65)	271 161 (51.47)
Male	213 430 (52.09)	39 959 (35.61)	2291 (46.35)	255 680 (48.53)
Age, y^a^				
<18	93 562 (22.83)	12 936 (11.53)	260 (5.26)	106 758 (20.26)
18-40	110 310 (22.92)	40 385 (35.99)	2374 (48.03)	153 069 (29.05)
40-65	129 628 (31.64)	46 410 (41.36)	1931 (39.07)	177 969 (33.78)
>65	93 377 (22.79)	19 644 (17.51)	562 (11.37)	113 543 (21.56)
Risk score				
Low (<9)	373 176 (91.08)	95 393 (85.02)	4683 (94.74)	473 252 (89.82)
Medium (9-16)	27 535 (6.72)	12 886 (11.48)	149 (3.01)	40 570 (7.70)
High (>16)	6155 (1.50)	3186 (2.84)	32 (0.65)	9373 (1.78)
Insurance type^b^				
High Mark, Blue Cross, WellSpan Pop Health	150 806 (36.81)	51 806 (46.17)	2387 (48.29)	204 999 (38.91)
Medicare	94 241 (23.00)	23 669 (21.10)	597 (12.08)	116 507 (22.49)
Medicaid	82 430 (20.12)	27 544 (24.55)	703 (14.22)	110 677 (21.01)
Other commercial	76 453 (18.66)	26 201 (23.35)	1158 (23.43)	103 812 (19.70)
Government	7672 (1.87)	2301 (2.05)	98 (1.98)	10 071 (1.91)

^a^ Age values had a discrepancy of approximately 5% between cohorts since some ages were recorded in months and may not have accounted accurately in the EMR, and there are other ages that change over the quality measurement time frame.

^b^ SlicerDicer was only able to measure proportions of encounters associated with the financial payer class. These data were unable to be exported for regression analysis. Self-pay was also unable to be measured due to conflation with cost-sharing (copays and deductibles). These proportions should be interpreted as approximate given that patients may have switched payers within the study time frame. Notably with the blended group, this may be particularly problematic given that these patients received both types of care. For Medicare and Medicaid, there may be redundancy of patients who had both payer types. The most important takeaway and interpretation of payer data are the comparable distribution of percentages across exposure groups.

As shown in Table 2, patients with telemedicine exposure had comparably better performance in 11 of 16 quality measures with statistically significant differences: all testing-based measures (patients with cardiovascular disease [CVD] with lipid panel: APD, 7.04%; 95% CI, 5.95%-8.10%; *P* < .001; patients with diabetes with hemoglobin A_1c_ testing: APD, 5.14%; 95% CI, 4.25%-6.01%; *P* < .001; patients with diabetes with nephropathy testing: APD, 9.28%; 95% CI, 8.22%-10.32%; *P* < .001; and blood pressure control: APD, 3.55%; 95% CI, 3.25%-3.85%; *P* < .001) and all counseling-based measures (cervical cancer screening: APD, 12.33%; 95% CI, 11.80%-12.85%; *P* < .001; breast cancer screening: APD, 16.90%; 95% CI, 16.07%-17.71%; *P* < .001; colon cancer screening: APD, 8.20%; 95% CI, 7.65%-8.75%; *P* < .001; tobacco counseling and intervention: APD, 12.67%; 95% CI, 11.84%-13.50%; *P* < .001; influenza vaccination: APD, 9.76%; 95% CI, 9.47%-10.05%; *P* < .001; pneumococcal vaccination: APD, 5.41%; 95% CI, 4.85%-6.00%; *P* < .001; and depression screening: APD, 4.85%; 95% CI, 4.66%-5.04%; *P* < .001).

**Table 2.** Statistical Comparison of Quality Performance Based on Telemedicine Exposure

Category and measure	Patients, No. (%)		Absolute percentage difference (95% CI), %^a^	*P* value
	Office-only (n = 409 732)	Telemedicine-exposed (n = 117 142)		
Medication-based				
CVD receiving antiplatelet	22 506 (71.63)	6924 (64.92)	6.71 (5.45 to 7.98)	<.001
CVD receiving statin	26 810 (77.74)	11 797 (75.95)	1.79 (0.88 to 2.71)	.001
HF on β-blocker	13 604 (63.65)	5127 (62.45)	1.20 (−0.35 to 2.76)	.13
Diabetes receiving statin	31 424 (71.89)	14 646 (70.77)	1.12 (0.23 to 2.01)	.01
URI antibiotic stewardship	5255 (96.16)	4073 (94.11)	2.05 (1.17 to 2.96)	<.001
Testing-based				
CVD with lipid panel	16 269 (79.15)	6032 (86.19)	−7.04 (−8.10 to −5.95)	<.001
Diabetes, HbA_1c_ testing	14 950 (85.62)	7154 (90.76)	−5.14 (−6.01 to −4.25)	<.001
Diabetes, nephropathy testing	16 788 (73.28)	8528 (82.56)	−9.28 (−10.32 to −8.22)	<.001
BP control (<140 mm Hg systolic; <90 mm Hg diastolic)	140 235 (88.64)	44 201 (92.19)	−3.55 (−3.85 to −3.25)	<.001
Counseling-based				
Cervical cancer screening	106 062 (42.28)	51 907 (54.61)	−12.33 (−12.85 to −11.80)	<.001
Breast cancer screening	54 874 (49.23)	17 279 (66.12)	−16.90 (−17.71 to −16.07)	<.001
Colon cancer screening	130 475 (49.23)	38 922 (39.56)	−8.20 (−8.75 to −7.65)	<.001
Tobacco counseling and intervention	44 787 (29.61)	18 189 (42.28)	−12.67 (−13.50 to −11.84)	<.001
Influenza vaccination	408 020 (20.00)	117 273 (29.76)	−9.76 (−10.05 to −9.47)	<.001
Pneumococcal vaccination	92 054 (12.18)	19 851 (17.60)	−5.41 (−6.00 to −4.85)	<.001
Depression screening	311 508 (2.10)	73 889 (6.95)	−4.85 (−5.04 to −4.66)	<.001

Abbreviations: BP, blood pressure; CVD, cardiovascular disease; HF, heart failure; HbA_1c_, hemoglobin A_1c_; URI, upper respiratory infection.

^a^ Absolute percent differences were determined with χ^2^ tests. For absolute differences, the χ^2^ test is in reference to the office-only group, thus positive values in this column indicate better performance for patients with office-only visits and negative values favor patients with telemedicine exposure.

Patients with office-only care had better performance in all the medication-based measures, but only 3 of the differences were statistically significant (patients with CVD receiving antiplatelets: APD, 6.71%; 95% CI, 5.45% to 7.98%; *P* < .001; patients with CVD receiving statins: APD, 1.79%; 95% CI, 0.88% to 2.71%; *P* = .001; and antibiotic stewardship for upper respiratory infections: APD, 2.05%; 95% CI, 1.17% to 2.96%; *P* < .001). There was no difference in quality performance for patients with heart failure receiving β-blockers (APD, 1.20%; 95% CI, −0.35% to 2.76%; *P* = .13) or patients with diabetes receiving statins (1.12%; 95% CI, 0.23% to 2.01%; *P* = .01).

Regression analysis revealed little to no difference after adjusting for demographic factors, except for the depression screening. Patients with telemedicine exposure had twice the odds of receiving screening (eTable 3 in the Supplement)

## Discussion

Compared with patients who had only office visits during the 18-month time frame, patients with telemedicine exposure had comparable or better performance in most quality measures we evaluated. Notwithstanding the statistical significance, the clinical relevance of these findings is perhaps more meaningful at the population health level for evaluating the outcomes of adding telemedicine as a care venue. Moreover, telemedicine exposure (especially blended office and telemedicine care) likely simulates a likely real-life scenario for the health consumer. Practically, these findings provide reassurance for health entities seeking to add telemedicine to their care capacity without reducing quality of care. And as we found, embracing telemedicine for enhancing certain aspects of care might be an avenue for enhancing quality performance in primary care.

Our findings facilitate an opportunity to evaluate telemedicine’s optimal role for improving primary care by analyzing the association of telemedicine exposure with quality performance. The favorability of prevention-based performance measures is particularly beneficial for health systems operating in value-based care models^38^ given that telemedicine has shown to improve health care costs^6,7,8^ and preventive health care efforts have an unforeseen feed-forward benefit in value-based care. Variability in telemedicine quality performance has been shown to depend on the clinical arena, but many studies have been narrow in application and scope.^5,19,39^ Our study adds a broader snapshot of telemedicine’s quality in primary care and evaluates the outcomes with standardized measures. Importantly, since the collection of practitioners in primary care specialties see the highest volume of patients nationally,^40^ this collective group has the highest prospect for preventing costly and preventable disease.^41^ Thus, it is paramount to understand when and where telemedicine is most clinically valuable, ie, what is telemedicine's optimal role.

Broadly, we found it helpful to consider findings within 2 main frameworks: (1) clinical intervention type (testing, medication, or counseling) and (2) outcome of telemedicine exposure (helping vs harming quality performance). First, regarding clinical intervention type (Table 2), we found modestly better quality performance in the office-only patients for medication-based measures (patients with CVD receiving antiplatelets or statins, patients with heart failure receiving β-blockers, patients with diabetes receiving statins, and antibiotic stewardship for upper respiratory infection). But for testing-based measures (CVD with lipid panel, diabetes with hemoglobin A_1c_ and nephropathy testing) and counseling-based measures (blood pressure control; cervical, breast, and colon cancer screening; tobacco screening; vaccination compliance; and depression screening), we found moderately better performance in the telemedicine-exposed group.

Second, regarding the outcomes of exposure, patients with telemedicine exposure performed comparatively better in most of the measures (11 of 16). Moreover, for 2 of the 5 measures in which office-only patients performed better, the differences were not statistically significant, indicating no difference in quality performance between groups in these measures. Thus, for 13 of 16 measures, patients with telemedicine exposure performed comparably or better than office-only patients. We interpret this as telemedicine probably augmenting quality performance or, more importantly, not harming it. Compared with zero exposure (office-only care), the telemedicine-exposed group had significantly better quality performance in important domains of chronic disease management and prevention, performing better or not differently in all diabetes measures and best in counseling-based measures (Table 2). This highlights the clinical domains where telemedicine could be considered an appropriate alternative venue to office-based care. In addition, for counseling-based measures like depression screening, telemedicine might even be a preferred venue, especially given that similar favorable outcomes are consistent with the literature.^42,43,44^

There may be clinical scenarios in which telemedicine is not the preferred venue. For example, this point is highlighted in the 3 instances in which performance in the office-only group was better than in the telemedicine-exposed group (antiplatelet therapy, statin therapy for heart disease, and antibiotic stewardship for upper respiratory infection). Nonetheless, in these comparisons, the quality performance difference was modest (2%-7%). This variation in performance difference might also infer minimal clinical effect or unmeasured patient experience effect. Regardless, our interpretation is still an overarching comparability between telemedicine and office-only quality of care, but this must be in the context of a known rural and urban divide (favoring urbanicity^45^), age and race preferences, medical severity of the population, and consideration of the pandemic era. However, the takeaway for health care leaders is that adding telemedicine poses low risk for negative outcomes for quality performance (except perhaps for select measures in cardiovascular care) and poses a potential for positive outcomes.

It is uncertain why the telemedicine-exposed group performed best overall. A reasonable explanation might be more opportunity to facilitate favorable quality outcomes. Telemedicine can potentially augment the encounter volume per patient, allowing the clinician multiple attempts to engage in quality measure–promoting intervention. An important consideration for this higher performance should include the type of clinical intervention. Most of the medication-based measures were in the cardiovascular domain (antiplatelets therapy, β-blockers, or statins), and it seems reasonable that these measures could perform better with an in-person interaction. Unlike the process-driven nature of measures that performed better in telemedicine-exposed patients, eg, testing-based measures (completing lipid panel, testing hemoglobin A_1c_ levels, or nephropathy testing) or counseling-based measures (cancer and depression screening, tobacco cessation, vaccinations), starting a medication, especially a life-long medication, could be a much bigger patient-clinician decision and thus better suited in more formal, in-person discussion. Furthermore, a reinforcing aspect of patient engagement, namely nonverbal communication, probably plays an important role in the success of these in-person, medication-based interactions. But perhaps for a large portion of chronic disease management and preventive care, telemedicine provides a more convenient, more accessible, and equally effective means of primary care.

Future studies could provide more granularity on optimizing the specific role of telemedicine in clinical scenarios, eg, understanding whether there is an association between stages of hypertension and effect modification attributable to the management venue or an association between venue and number of blood pressure medications. This would provide insight on where to invest in health care infrastructure and what clinical venue would be most valuable. This could also guide venue selection for patients initiating antihypertensive therapy vs patients requiring a third antihypertensive. Such insight would promote win-win environments to increase value: improved health outcomes for patients and incentive for clinicians and health systems operating in value-based care models.

For health policy surrounding telemedicine funding, this study reinforces the described need for high-value care after the pandemic.^46^ As Medicare waivers have temporarily facilitated the reimbursement of telemedicine during the pandemic,^24^ the medical literature needs more support in a post–Coronavirus Aid, Relief, and Economic Security Act world where many patients relying on telemedicine could lose access to health care.^47^ Our study is timely in addressing these quality concerns; it is no longer a hopeful question—can we deliver quality care with telemedicine?—but an evidence-supported statement: we can deliver quality care with telemedicine.

### Limitations

This study has limitations. First, we were unable to control for the number of visits between office-only and telemedicine-exposed groups. Building accurate quality measures in SlicerDicer required a patient data model (mining data by number of patients instead of number of visits). Similarly, given the deidentified aggregate data, it was not possible to uniquely verify visit numbers (this would have required unique patient identifiers and a breach of institutional review board constraints). Furthermore, although we attempted to simulate a graded response to telemedicine exposure (blended and telemedicine-only subgroups), we could only reliably evaluate visit type binarily, distinguishing only whether a patient had telemedicine visits, not the percentage thereof. The ability to evaluate telemedicine exposures as a continuous variable or a marginal average effect over graded levels would provide insight on the ideal blend of telemedicine and facilitate stronger conclusions. This insight highlights a gap in the literature.

Second, we recognize known limitations of the EMR. SlicerDicer is a powerful data mining tool with published methods (including COVID-19 literature^48,49^), but we were reliant on clinicians accurately updating medical history, patient problem lists, and medication reconciliation and documenting counseling. Additionally, testing or procedures performed outside the health system were unaccounted for, possibly underrepresenting true quality performance. We also would have liked to compare telemedicine (video) vs telephone (nonvideo) vs in-person office visits, but as noted in the Methods section, telephone encounters conflated nonclinician visits and compromised the validity of quality analysis. Furthermore, we could not measure variability of the digital divide^50^ in our population to observe any association with internet access for telemedicine visits.

Additionally, we recognize sampling limitations. Although large national data sets such as Medicare claims were desirable for this study, these claims-based data are historical on acquisition, making it difficult to generate a timely snapshot of quality. Further, distinguishing telemedicine visits from claims-based evaluation and management codes data are difficult, we were not successful in this approach even within our own health system’s billing department. We recognize that using a single health system data set introduces sample bias but felt the timeliness and applicability of findings could outweigh sample size limitations.

## Conclusions

This cohort study found early evidence of telemedicine’s favorable association with the quality of primary care during COVID-19. For chronic disease management and preventive care, telemedicine exposure appeared to have had a positive association with HEDIS quality performance, and this study highlights a gap in the literature: understanding the relationship between the ideal blend of telemedicine and in-office care. For policy makers, these findings of comparable quality support telemedicine’s continued funding. For practices and health systems, this study demonstrates telemedicine’s value in appropriate populations: augmenting primary care capacity without negatively affecting care quality.

## Notes

Supplement. **eAppendix.** Supplemental Methods **eTable 1.** Comparison of 16 HEDIS Performance Measures Between Historical Baseline and Study Timeframe **eTable 2. **Description of Quality Measure Details and Data Mining Specifications for Performance Measures **eTable 3.** Percentage of Patients Encounter Meeting HEDIS Quality Measure Criteria by Encounter Type (Adjusted and Nonadjusted)
eReferences.
