# Supplementary material for: Comparison of Quality Performance Measures for Patients Receiving In-Person vs Telemedicine Primary Care in a Large Integrated Health System
Source: JAMA Netw Open. 2022 Sep 26;5(9):e2233267. doi: 10.1001/jamanetworkopen.2022.33267 (PMC9513647; doi:10.1001/jamanetworkopen.2022.33267)
Supplement: Supplement. — eAppendix. Supplemental Methods eTable 1. Comparison of 16 HEDIS Performance Measures Between Historical Baseline and Study Timeframe eTable 2. Description of Quality Measure Details and Data Mining Specifications for Performance Measures eTable 3. Percentage of Patients Encounter Meeting HEDIS Quality Measure Criteria by Encounter Type (Adjusted and Nonadjusted) eReferences. [file jamanetwopen-e2233267-s001.pdf]

Supplemental Online Content

Baughman DJ, Jabbarpour Y, Westfall JM, et al. Comparison of quality performance measures for patients receiving in-person vs telemedicine primary care in a large integrated health system. *JAMA Netw Open.* 2022;5(9):e2233267. doi:10.1001/jamanetworkopen.2022.33267

eAppendix. Supplemental Methods

eTable 1. Comparison of 16 HEDIS Performance Measures Between Historical Baseline and Study Timeframe

eTable 2. Description of Quality Measure Details and Data Mining Specifications for Performance Measures

eTable 3. Percentage of Patients Encounter Meeting HEDIS Quality Measure Criteria by Encounter Type (Adjusted and Nonadjusted)

eReferences.

This supplemental material has been provided by the authors to give readers additional information about their work.

© 2022 Baughman DJ et al. *JAMA Network Open.*

eAppendix. Supplemental Methods

SlicerDicer Data Mining

Using a standardized, general approach to measure numerators and denominators in SlicerDicer provided consistent results. This necessitated 32 unique data sessions to ensure accurate numerators and denominators: 16 for office-only patients and 16 for telemedicine exposed patients. First, we built the HEDIS specified denominator criteria and noted the total number of patients. Next, we added the numerator criteria (unique from the denominator criteria), this resulted in a smaller proportion of the denominator. These numerator filters ensured that we were working within the same population between cohorts. We then exported deidentified patient data into excel to use automated calculations of differences between cohorts and sub-groups. We analyzed excel calculated percentages with SlicerDicer percentages to ensure comparability. Importantly, this approach avoided reliance on unreliable or inaccurate data representation within SlicerDicer.

To ensure unique denominators for patients in the divided cohort, we used a patient data model that counted the number of patients (rather than number of visits). Importantly, this avoided redundant inclusion (cross contamination) of the same patient between denominator groups. Moreover, since we built separate data sessions for the divided cohort, redundant numerator counts between groups (office-only versus telemedicine-exposed) was not possible. Appropriately, this avoided double counting quality performance between groups, especially important for patients that had both office and telemedicine encounters (these patients for example, would only be counted in the telemedicine-exposed group since having a telemedicine encounter would exclude them from the office-only group).

Patient data models were used in 16 separate sessions to ensure accuracy in data mining. We used three primary data filters in every session to ensure consistency (these filters were all “linked”): (1) “primary care service line” (limiting the total number of patients to only primary care encounters), (2) “face-to-face encounter” (limiting to only live encounters with clinicians, filtering out any telephone triage or medication refill encounters), (3) “encounter type” (office-only, telemedicine-only, or office and telemedicine). For the “encounter type” filter, 3 sequences were conducted for each of the 16 measures to capture cohort numerators and denominators between groups: first, an office subfilter with exclusion of telemedicine; second, a telemedicine subfilter with exclusion of office; third, office and telemedicine combined.

Historical (pre-pandemic) baseline

This was the methodological approach to constructing the historical baseline comparison to the study timeframe. As shown in table 1, there was largely comparable quality performance by patients with identical selection criteria (see manuscript methods, figure 1) from pre-pandemic to pandemic. Further, the majority of differences between timeframes was less than 5%. It was important to avoid case-mix adjustments or timeframe selections in our data set since the NQF states that this can impair validity and reliability of results[1,2]. So evaluating the population over nearly a 4 year timeframe provided further reassurance to results of the study.

Quality measure selection

Of the 23 total measures in the original CQMC data set for primary care[3], we ultimately chose only 16 measures since obtaining all 23 was not possible with the limitations of our EMR data extraction methods. We excluded measures that were either (1) unable to be tracked within the EMR (for example, diabetic eye exams that were done outside the health system), (2) difficult to obtain (for example, BMI and alcohol use counseling, medicine reconciliations, or patient experience surveying), or (3) too complex to obtain within the study timeframe (for example, HbA1c <9 or asthma control which involves complex calculations of medication ratios). As mentioned in the methods of the manuscript, measures from CMS

© 2022 Baughman DJ et al. *JAMA Network Open.*

were also added. Appendix table 2 reveals these details in the "measure steward" with any adjustments or additions made to each of NQF's quality measures[2].

A notable adjustment was made to the nephropathy measure for patients with diabetes. For nephropathy evaluation as a HEDIS measure, the literature has described the overestimation and subsequent overreporting of reporting compliance of nephropathy screening when the measure numerator includes the presence of ACEi/ARBs prescription (inferring microalbuminuria from the presence of these medications may not be reliable)[4]. Thus, authors chose to not include the presence of ACEi/ARB in the numerator and only measured nephropathy tests (microalbuminuria, UA or protein/creatinine ratio).

Technical Appendix

EHR data from a large integrated non-profit healthcare system was used to compare in-office and telemedicine outpatient encounters. Primary care quality measures from 3/1/20 to 4/30/21 were evaluated and analyzed separately for each of these cohorts so that measures were linked to corresponding encounter types.

Of the sixteen-quality metrics, four measures were used as outcomes in regression analyses (statin therapy, flu vaccination, blood pressure control and depression screening)**.** We selected these measures from each quality domain based on the highest encounter count. The receipt of statin therapy for patients with diabetes among adults 40-75 years and who do not have clinical ASCVD, was coded as '1' if they received and adhered to statin therapy, otherwise codes as '0'. For the high blood pressure control measure, adults 18-85 years of age who had a diagnosis of hypertension (HTN) and whose blood pressure was adequately controlled (<140/90 mm Hg) during the measurement year were coded as '1', and 'o' for those with uncontrolled blood pressure.

Patients aged 6 months and older seen for a visit between October 1 and March 31 who received an influenza immunization OR who reported previous receipt of an influenza immunization were coded as '1' and '0' if they did not receive it. For depression screening measure patients aged 12 years and older screened for depression on the date of the encounter or 14 days prior to the date of the encounter using an age-appropriate standardized depression screening tool AND if positive, a follow-up plan documented on the date of the eligible encounter were coded as '1' and '0' otherwise.

The type of visit (telehealth or office-based encounter) was used as the explanatory variable. Age in years as a continuous measure, binary gender (male or female), race (White, Black or African American, Other, Asian, American Indian or Alaska Native, Native Hawaiian or Other Pacific Islander), ethnicity (Hispanic and Not Hispanic or Latino), and overall adult risk scores were used as controls. Binary logistic regression models were performed separately for each of the selected outcomes using Stata 16.0 (Stata Corp). The alpha was set at $p<0.05$).

For all the regression models, Odds Ratios, 95% Confidence Interval and p-values were reported (Appendix Table 3).

© 2022 Baughman DJ et al. *JAMA Network Open.*

eTable 1. Comparison of 16 HEDIS Performance Measures Between Historical Baseline and Study Timeframe

HEDIS Measures		Pre-pandemic (1/1/18-2/29/20) HEDIS quality performance (%) *(n=total eligible = denominator)*				Study time frame (3/1/20 to 11/30/21) HEDIS quality performance (%) *(n=total eligible = denominator)*			
Brief description		Total	Office-only	Telemedicine exposed		Total	Office-only	Telemedicine exposed	
				Blended	Telemedicine-only			Blended	Telemedicine-only
**Cardio-vascular Care**	% with HTN and BP <140/90	94.33% (170,537)	94.33% (170,040)	97.70% (478)	21.05% (19)	89.49% (184,436)	88.64% (140,235)	94.48% (42,490)	35.18% (1,711)
	% with CVD on antiplatelet therapy	64.02% (25,655)	64.06% (25,613)	39.02% (41)	100% (1)	**70.05% (29,430)**	71.63% (22,506)	65.08% (6,762)	58.02% (162)
	% with CVD or MI on a statin*	71.79% (32,585)	71.80% (32,514)	65.71% (70)	100% (1)	**77.20% (38,607)**	77.74% (26,810)	75.99% (11,618)	73.74% (179)
	% with CVD and lipid testing	85.37% (18,955)	85.33% (18,896)	98.28% (58)	100% (1)	81.05% (22,301)	79.15% (16,269)	86.78% (5,922)	54.55% (110)
	% with HF on Beta Blocker Therapy*	61.60% (12,855)	61.62% (12,836)	47.37% (19)	0.00% (0)	**63.32% (18,731)**	63.65% (13,604)	62.57% (95,039)	0.00% (88)
**Diabetes**	% with DM and HbA1c testing	91.85% (23,236)	91.83% (23,160)	98.67% (75)	100% (1)	87.28% (22,104)	85.62% (14,950)	91.68% (6,863)	69.07% (291)
	% with DM and nephropathy testing	83.34% (22,866)	83.35% (22,791)	79.73% (74)	100% (1)	76.41% (25,316)	73.28% (16,788)	83.26% (8,376)	44.08% (152)
	% with DM on a statin	56.78% (39,675)	56.81% (39,548)	48.39% (124)	33.33% (3)	**71.54% (46,070)**	71.89% (31,424)	70.96% (14,362)	61.27% (284)
**Prevention & Wellness**	% with appropriate Cervical Cancer Screening	47.02% (142,309)	46.80% (140,969)	71.09% (1,304)	52.78% (36)	46.33% (157,969)	42.28% (106,062)	55.26% (49,879)	38.51% (2,028)
	% patients with biennial mammography	34.25% (64,742)	34.06% (64,463)	78.39% (273)	50.00% (6)	**53.27% (72,153)**	49.23% (54,874)	67.13% (16,671)	38.32% (608)

© 2022 Baughman DJ et al. *JAMA Network Open.*

	% with appropriate CC screening	31.00% (150,693)	31.02% (150,271)	23.19% (414)	25.00% (8)	**33.24%** **(**169,397)	31.36% (130,475)	40.16% (37,640)	22.07% (1,282)
	% of smokers with counseling or cessation meds	27.86% (56,575)	27.84% (56,420)	33.11% (151)	0.00% (4)	**33.27%** **(**62,976)	29.61% (44,787)	42.90% (17,461)	27.47% (728)
	% with influenza vaccination	22.56% (469,169)	22.41% (466,998)	55.63% (2,114)	63.16% (57)	22.18% (525,293)	20.00% (408,020)	30.39% (112,346)	15.32% (4,927)
	% with pneumococcal vaccination	25.66% (95,500)	25.65% (95,423)	40.79% (76)	100% (1)	13.14% (111,905)	12.18% (92,054)	17.95% (19,316)	4.86% (535)
**Behavioral health**	% screened for depression	2.09% (341,931)	0.74% (340,495)	4.04% (1,386)	18.00% (50)	**3.03%** **(**385,397)	2.10% (311,508)	7.17% (69,899)	3.18% (3,990)
**Pulmonary**	% with acute bronchitis NOT prescribed antibiotics	94.78% (25,059)	94.82% (24,766)	91.44% (292)	100% (1)	**95.26%** **(**9,328)	96.16% (5,255)	94.08% (4,017)	96.43% (56)

Abbreviations: BP = blood pressure, HTN = hypertension, CVD = cardiovascular disease, MI =myocardial infarction, HF = heart failure, DM = diabetes mellitus, CC = colorectal cancer
For column headers: "office" includes patients seen only in the office setting (excludes patients with any telemedicine encounters), "blended" includes only patients with office and telemedicine encounters (having at least one of each encounter type during the timeframe), and telemedicine includes only patients seen via video telemedicine (excludes patients with any office encounters).

Overall, in the pre-COVID-19 timeframe (historical baseline), interpretation of blended and telemedicine-only HEDIS performance should be in the context of the very small volume of telemedicine encounters throughout the health system. HEDIS numerators (not shown) were calculated according to the measure steward specifications (see appendix). Bolded values in the "total" column during the study timeframe indicate where quality performance increased compared to the historical baseline (improved in 9 of 16 measures).

© 2022 Baughman DJ et al. *JAMA Network Open.*

eTable 2. Description of Quality Measure Details and Data Mining Specifications for Performance Measures

Measure Utilized	Measure Steward	SlicerDicer specifications	Measure descriptions
**Cardiovascular**			
Blood pressure			
Percentage of population with controlled BP	NCQA & CPC/CPC+	Diagnosis filter: Hypertension (grouper), face-to-face encounter, primary care service line<br><br>Age: males 18-85 years<br><br>Measure: Percentage of BP <140/90 (by population before and after, not measure)<br><br>*Exclude* (hospice, pregnancy, ESRD, dialysis)	Controlling High Blood Pressure: The percentage of adults 18-85 years of age who had a diagnosis of hypertension (HTN) and whose blood pressure was adequately controlled (<140/90 mm Hg) during the measurement year[5].<br><br>Numerator Statement: Patients whose most recent blood pressure level was <140/90 mm Hg during the measurement year.<br><br>Denominator Statement: Patients 18-85 years of age who had at least two visits on different dates of service with a diagnosis of hypertension during the measurement year or the year prior to the measurement year.<br><br>Excludes: Adults in hospice. It also excludes adults with advanced illness and frailty, as well as Medicare adults 65 years of age and older enrolled in an I-SNP or living long-term in institutional settings. Additionally, this measure excludes patients with evidence of end-stage renal disease, dialysis, nephrectomy, or kidney transplant on or prior to December 31 of the measurement year. It also excludes female patients with a diagnosis of pregnancy during the measurement year, and patients who had a nonacute inpatient admission during the measurement year.
Statin Therapy			
CVD patients on statin therapy	NCQA & CPC/CPC+	Diagnosis filter: ischemic vascular disease (grouper) OR Heart Disease (grouper)<br>face-to-face encounter, primary care service line<br><br>Age: males 21–75 years<br>Age: females 40–75 years<br><br>Measure: Statins pharm class	Statin Therapy for Patients with Cardiovascular Disease[6]: Assesses males and females who have clinical atherosclerotic cardiovascular disease (ASCVD) and who received and adhered to statin therapy.<br><br>Numerator Statement: Adults with ASCVD who have received and adhered to statin therapy.<br><br>Denominator Statement: Males 21–75 years of age and females 40–75 years of age who have clinical ASCVD and had a visit with the primary care provider
Ischemic Vascular Disease			
CVD patient on antiplatelet therapy	CPC/CPC+	Diagnosis filter: IVD (multiple QM-groupers), CAD grouper, Acute MI grouper, face-to-face encounter, primary care service line<br><br>Age: 18 and older (Male & Female)<br><br>Measure: Antiplatelet pharm class<br><br>*Exclude:* hospice or anticoagulation therapy	Ischemic Vascular Disease (IVD): Use of Aspirin or An Antithrombotic[7]<br><br>Numerator Statement:<br>Patients who had documentation of routine use of aspirin or an antiplatelet during the measurement year.<br><br>Denominator Statement:<br>Patients 18 years or older by the end of the measurement year discharged from an inpatient setting with an AMI, CABG, or PCI during the 12 months prior to the measurement year or who had a diagnosis of IVD during both the measurement year and the year prior to the measurement year.<br><br>Denominator Exclusion(s): Patients who had documentation of use of anticoagulant medications overlapping the measurement year Exclude patients who were in hospice care during the measurement year. [3]

© 2022 Baughman DJ et al. *JAMA Network Open.*

Lipid panel was performed	CMS, CPC/CPC+	Diagnosis: IVD, CHD or heart disease grouper (unlinked), face-to-face encounter, primary care service line<br><br>Measure: before and after of having lab test or lab component (unlinked)<br><br>*Exclude:* pregnancy or on palliative care	Preventive Care and Screening: Cholesterol - Fasting Low Density Lipoprotein (LDL-C) Test Performed (eCQM)[8]<br><br>Numerator Statement:<br>Patients who had a fasting LDL-C test performed or a calculated LDL-C during the measurement period<br><br>Denominator Statement:<br>All patients aged 20 through 79 years who have CHD or CHD Risk Equivalent OR 10-Year Framingham Risk > 20%<br><br>Exclusions: Patients who have an active diagnosis of pregnancy OR Patients who are receiving palliative care
Heart Failure			
Ongoing Beta Blocker Therapy for Left Ventricular Systolic Dysfunction	2019: MIPS CQMS<br><br>CPC/CPC+	Diagnosis: Heart failure (grouper), unlinked, face-to-face encounter, primary care service line<br><br>Age: 18-85<br><br>Measure: Percentage on a BB	Percentage of patients aged 18 years and older with a diagnosis of heart failure (HF) with a current or prior left ventricular ejection fraction (LVEF) < 40% who were prescribed beta-blocker therapy either within a 12-month period when seen in the outpatient setting or alternatively at each hospital discharge[9]<br><br>Numerator Statement: Patients who were prescribed beta-blocker therapy within a 12-month period when seen in the outpatient setting<br><br>Denominator Statement: All patients aged 18 years and older with a diagnosis of heart failure with a current or prior LVEF < 40%.
**Diabetes**			
DM testing			
Hba1c testing	NCQA	Diagnosis: T1 or T2 DM (unlinked), primary care service line<br><br>Age: 18-75<br><br>Measure: Percentage of patients with an A1c "final" lab component during measurement period	Assesses adults 18–75 years of age with diabetes (type 1 and type 2) who had Hemoglobin A1c (HbA1c) testing[5,10].<br><br>Numerator Statement:<br>Patients who had an HbA1c test performed during the measurement year.<br><br>Denominator Statement:<br>Patients 18-75 years of age by the end of the measurement year who had a diagnosis of diabetes (type 1 or type 2) during the measurement year or the year prior to the measurement year.<br><br>Exclude: patients who use hospice services[2].
Nephropathy			
Urinalysis or Protein/Creatinine ratio testing	NCQA & CPC/CPC+	Diagnosis: T1 or T2 DM (unlinked), face-to-face encounter, primary care service line<br><br>Age: 18-75<br><br>Measure: Lab component (Prot/Cr, microalbumin + HEDIS measure)<br><br>*Exclude: palliative or hospice care*	Measure Description:<br>The percentage of patients 18-75 years of age with diabetes (type 1 and type 2) who received a nephropathy screening test or monitoring test or had evidence of nephropathy during the measurement year.<br>Microalbuminuria, Urinalysis, or protein/creatinine ratio[2]<br><br>Numerator Statement: Patients receiving a nephropathy screening or monitoring test or having evidence of nephropathy during the measurement year<br><br>Denominator Statement:<br>Patients 18-75 years of age by the end of the measurement year who had a diagnosis of diabetes (type 1 or type 2) during the measurement year or the year prior to the measurement year.<br>Exclusions: patients who use hospice services.

© 2022 Baughman DJ et al. *JAMA Network Open.*

			**Note**: "on ACEi/ARB" was not included in this measurement (not to be confused with excluded, see above explanation in "quality measure selection")[4]
Statin therapy			
DM patients on statin therapy	NCQA & CPC/CPC+	Diagnosis: T1 or T2 DM (unlinked), face-to-face encounter, primary care service line<br><br>Age: 40-75<br><br>Measure: pharmaceutical class-HMGCoA reductase inhibitors (statins)	Statin Therapy for Patients with Diabetes: Assesses adults 40-75 years of age who have diabetes and who do not have clinical ASCVD, who received and adhered to statin therapy.
**Prevention & Wellness**			
Cervical Cancer Screening			
Percentage of females screened	NCQA & CPC/CPC+	Population: females 21-64, face-to-face encounter, primary care service line<br><br>Measure(s): before/after pop filter:<br>1. procedure: HPV or reflex testing, age 30-64, range 3/1/15 to 4/30/21 (5 year range)<br>2. procedure: paps only, age 21-64, range 3/1/17 to 4/30/21 (3 years)<br><br>*Exclude:* hysterectomy	Cervical Cancer Screening: The percentage of women 21–64 years of age who were screened for cervical cancer using either of the following criteria[2]:<br>• Women 21–64 years of age who had cervical cytology performed within the last 3 years.<br>• Women 30–64 years of age who had cervical high-risk human papillomavirus (hrHPV) testing performed within the last 5 years.<br>• Women 30–64 years of age who had cervical cytology/high-risk human papillomavirus (hrHPV) co-testing within the last 5 years.<br>Numerator Statement: The number of women who were screened for cervical cancer.<br><br>Denominator Statement: Women 24-64 years of age as of the end of the measurement year.<br><br>Exclusions: Hysterectomy with no residual cervix, cervical agenesis or acquired absence of cervix any time during their medical history through the end of the measurement year.
Breast Cancer Screening			
Percentage of biennial mammography screening	NCQA & CPC/CPC+	Population: females 50-74, face-to-face encounter, primary care service line<br><br>Measure(s): Percentage of population getting mammogram procedure in the prior 2 years*<br><br>*per USPSTF = biennial screening<br><br>*Exclude:* hospice or palliative care	Breast Cancer Screening: Percentage of women 50-74 years of age who had a mammogram to screen for breast cancer[2]<br><br>Numerator Statement: Women who received a mammogram to screen for breast cancer.<br><br>Denominator Statement: Women 50-74 years of age.<br><br>Excludes: history of bilateral mastectomy, use of hospice services or are enrolled in an institutional special needs plan or living long-term in an institution any time during the measurement year.
Colorectal Cancer Screening			
Percentage of patients who received appropriate Colon Cancer screening	NCQA & CPC/CPC+ USPSTF	Population: age 51-75 + face-to-face encounter, primary care service line	Colorectal Cancer Screening (COL): The percentage of patients 50–75 years of age who had appropriate screening for colorectal cancer[2,11]<br><br>Numerator Statement: Patients who received one or more screenings for colorectal cancer according to clinical guidelines.

© 2022 Baughman DJ et al. *JAMA Network Open.*

		Measure(s): before/after to capture both*<br>1. lab components: hemoccult, FIT (DNA + groupers)<br>2. procedures: colonoscopy groupers, CT colonography), Flexible sigmoidoscopy<br><br>**subfilters for appropriate testing time frame guidelines*<br><br>*Exclude:* colectomy, hospice, colon cancer/colectomy exclusion (grouper), palliative grouper	Denominator Statement: Patients 51–75 years of age<br><br>Excludes: patients with a history of colorectal cancer or total colectomy, patients who use hospice services or are enrolled in an institutional special needs plan (SNP) or living long-term in an institution any time during the measurement year.<br><br>Appropriate testing timeframes:<br>1. Annual fecal occult blood or FIT testing<br>2. Triennial sDNA-FIT testing<br>3. Colonoscopy or flexible sigmoidoscopy with FIT every 10 years
Tobacco Use: Screening and Cessation			
Patients screened within last 1 year<br>(*not 2 years as there was not enough telemedicine data*)	NCQA & CPC/CPC+	Population: age greater than 18 (screening), primary care service line, nicotine dependence (F17* ICD-10), tobacco use (Z72.0 ICD-10), Tobacco user [grouper]<br><br>Measure: (as below)	Preventive Care and Screening: Tobacco Use: Screening and Cessation Intervention: Percentage of patients aged 18 years and older who were screened for tobacco use one or more times within 24 months AND who received tobacco cessation intervention if identified as a tobacco user[4]<br><br>Three rates are reported:<br>a. Percentage of patients aged 18 years and older who were screened for tobacco use one or more times within 24 months.<br><br>Exclude: limited life expectancy, other medical reason)
Counseling and cessation efforts carried out for patients		Patients aged 18 years and older, tobacco user<br><br>face-to-face encounter, primary care service line<br><br>Measure: Smoking Adherence [pharma class] OR Z71* ICD-10 [grouper] OR received tobacco cessation intervention OR screened for tobacco use AND received tobacco cessation intervention<br><br>*Exclude: limited life expectancy*	Population: age >18, tobacco use OR nicotine use, smoker and:<br>1. Percentage who received cessation counseling<br>2. Percentage who were prescribed quitting meds<br><br>Exclude:<br>Limited life expectancy<br><br>Measure(s): before and after adding the counseling/deterrents
Influenza immunization			
Annual influenza vaccination rate	CPC/CPC+	Age: greater than 6 months, face-to-face encounter, primary care service line<br><br>Measure:<br>Sequential measure - received within last 1 year (due to seasonality), since 10/1/2019<br>(true for the Oct-Mar timeframe per CMS measure)	Percentage of patients aged 6 months and older seen for a visit between October 1 and March 31 who received an influenza immunization OR who reported previous receipt of an influenza immunization[12]<br><br>Numerator: Patients who received an influenza immunization OR who reported previous receipt of an influenza immunization<br><br>Denominator: All patients aged 6 months and older seen for a visit during the measurement period<br><br>Exclude: Influenza immunization was not administered for reasons documented by clinician (e.g., patient allergy or other medical reasons, patient declined or other patient reasons, vaccine not available or other system reasons) (G8483)

© 2022 Baughman DJ et al. *JAMA Network Open.*

		Exclude: limited life expectancy	
Pneumococcal vaccination			
Patients for whom vaccine was ordered	CPC/CPC+	Population: >65 + face-to-face encounter, primary care service line<br><br>Measure: Patients who ever received the pneumococcal vaccine (not sequential)	Percentage of patients 65 years of age and older who have ever received a pneumococcal vaccine[13]<br><br>Numerator: Patients who have ever received a pneumococcal vaccination before the end of the measurement period<br><br>Denominator: Patients 65 years of age and older with a visit during the measurement period.
**Behavioral health**			
Depression screening			
Patients with depression who were screened with PHQ	CMS (NQF), CPC/CPC+	Population: all patients greater than the age of 12 or more<br><br>face-to-face encounter, primary care service line<br><br>*Excludes:* Depression and BP<br><br>Measure: before and after pop filter:<br>1. PHQ-2 or PHQ-9<br>2. documented plan = procedure orders (behavioral health grouper, ambulatory referral behavioral health)<br>within 1 the measurement year (i.e. the time frame)	Screening for Depression and Follow-Up Plan: Percentage of patients aged 12 years and older screened for depression on the date of the encounter or 14 days prior to the date of the encounter using an age appropriate standardized depression screening tool AND if positive, a follow-up plan is documented on the date of the eligible encounter[14]<br><br>The Institute for Clinical Systems Improvement (ICSI) = the CMS citation, says primary care<br><br>Numerator Statement:<br>Patients screened for depression on the date of the encounter or up to 14 days prior to the date of the encounter using an age-appropriate standardized tool AND, if positive, a follow-up plan is documented on the date of the eligible encounter<br><br>Denominator Statement:<br>All patients aged 12 years and older at the beginning of the measurement period with at least one eligible encounter during the measurement period<br><br>Exclusions<br>• Patient has an active diagnosis of depression prior to any encounter during the measurement period<br>• Patient has a diagnosed bipolar disorder prior to any encounter during the measurement period and does not have depression or bipolar disorder.
**Pulmonary**			
Avoidance of Antibiotics			
Patients with acute bronchitis and antibiotics were avoided	NCQA	Diagnosis: acute bronchitis and bronchitis (unlinked), primary care service line<br><br>Age: 18-64<br>face-to-face encounter, primary care service line<br><br>Measure: Received antibiotics in following 3 days	Avoidance of Antibiotic Treatment in Adults With Acute Bronchitis[15]<br><br>Numerator Statement:<br>Patients who were dispensed antibiotic medication on or three days after the index episode start date (a higher rate is better). The measure is reported as an inverted rate (i.e., 1- numerator/denominator) to reflect the number of people that were not dispensed an antibiotic.<br><br>Denominator Statement:<br>All patients 18 years of age as of January 1 of the year prior to the measurement year to 64 years as of December 31 of the measurement year with an outpatient or ED visit with any diagnosis of acute bronchitis during the Intake Period (January 1–December 24 of the measurement year).

© 2022 Baughman DJ et al. *JAMA Network Open.*

eTable 3. Percentage of Patients Encounter Meeting HEDIS Quality Measure Criteria by Encounter Type (Adjusted and Nonadjusted)

VARIABLES	Blood Pressure Control				Patients with diabetes on statins				Influenza vaccination				Depression screening			
	Unadjusted		Adjusted		Unadjusted		Adjusted		Unadjusted		Adjusted		Unadjusted		Adjusted	
	OR	95% CI	OR	95% CI	OR	95% CI	OR	95% CI	OR	95% CI	OR	95% CI	OR	95% CI	OR	95% CI
Telemedicine visits	1.046	1.028 - 1.064	1.045	1.027 - 1.063	0.937	0.886 - 0.991	1.059	0.999 - 1.123	1.23	1.211 - 1.249	1.182	1.164 - 1.201	2.145	2.032 - 2.264	2.029	1.921 - 2.143
Age in years			1.001	1.000 - 1.001			1.041	1.039 - 1.044			1.009	1.008 - 1.009			0.988	0.987 - 0.989
Gender: Female			1.028	1.014 - 1.042			0.716	0.684 - 0.749			1.096	1.084 - 1.109			1.148	1.095 - 1.203
Race: Black or African American			0.94	0.909 - 0.973			1.063	0.963 - 1.173			0.84	0.816 - 0.864			1.236	1.124 - 1.358
Race: Otherb			0.988	0.938 - 1.042			1.16	1.001 - 1.344			0.999	0.966 - 1.034			1.019	0.890 - 1.167
Race: Asianb			0.996	0.917 - 1.082			1.368	1.109 - 1.687			1.381	1.310 - 1.457			1.227	0.990 - 1.519
Race: American Indian or Alaska Nativeb			1.034	0.856 - 1.250			1.731	0.944 - 3.173			0.975	0.831 - 1.145			1.228	0.687 - 2.193
Race: Native Hawaiian or Other Pacific Islandeb			0.961	0.718 - 1.287			0.674	0.297 - 1.531			0.975	0.757 - 1.257			0.248	0.0347 - 1.777
Race: Hispanic or Latinoc			0.991	0.941 - 1.043			1.112	0.961 - 1.286			0.946	0.916 - 0.978			0.98	0.859 - 1.117
Race: Overall Adult Risk Score			1.01	1.009 - 1.012			1.067	1.061 - 1.072			1.025	1.023 - 1.026			1.114	1.109 - 1.120
Constant	0.880***	0.873 - 0.886	0.772***	0.746 - 0.800	2.958***	2.887 - 3.030	0.184***	0.158 - 0.213	0.355***	0.353 - 0.357	0.211***	0.208 - 0.214	0.0205***	0.0200 - 0.0210	0.0219***	0.0205 - 0.0234
Total Observations	335,563		335,563		42,731		42,731		615,866		615,866		324,224		324,224	

© 2022 Baughman DJ et al. *JAMA Network Open.*

Odds Ratios showing Uptake of Preventive Services by Type of Visit (In-office versus Telemedicine). Adjusted values revealed little to no difference in quality performance with the exposure of telemedicine, except for depression screening where patients with telemedicine encounters had twice the odds of having depression screening than the patients in-office encounter. Variables included all the factors in table 1 (except payer type).

a Compared to office-only visits
b Race compared to Whites*
c Ethnicity compared to "not hispanic or latino"

OR, Odds Ratios;; CI, Confidence Interval

***p<0.01, **p<0.05, *p<0.1*

© 2022 Baughman DJ et al. *JAMA Network Open.*

eReferences

1. NQF: Measuring Performance. Accessed March 20, 2021. https://www.qualityforum.org/Measuring_Performance/Measuring_Performance.aspx
2. NQF: Quality Positioning System ™. Accessed March 20, 2021. https://www.qualityforum.org/QPS/QPSTool.aspx?m=850&e=1#qpsPageState=%7B%22TabType%22%3A1
3. Core Quality Measures Collaborative. Core Quality Measures Collaborative. Accessed August 14, 2021. http://www.qualityforum.org/cqmc/
4. Krause TM, Ganduglia-Cazaban C, Finkel KW. Rates for HEDIS Screening for Diabetic Nephropathy Quality Measure May Be Overstated. *Manag Care*. 2018;27(8):45-49.
5. Consensus Core Set: ACO and PCMH/Primary Care. Published online October 9, 2020. http://www.qualityforum.org/WorkArea/linkit.aspx?LinkIdentifier=id&ItemID=88907
6. Statin Therapy for Patients With Cardiovascular Disease and Diabetes. NCQA. Accessed September 1, 2021. https://www.ncqa.org/hedis/measures/statin-therapy-for-patients-with-cardiovascular-disease-and-diabetes/
7. Ischemic Vascular Disease (IVD): Use of Aspirin or An Antithrombotic. CMS measures inventory tool. Accessed September 1, 2021. https://cmit.cms.gov/CMIT_public/ViewMeasure?MeasureId=692
8. Preventive Care and Screening: Cholesterol - Fasting Low Density Lipoprotein (LDL-C) Test Performed (eCQM). CMS measures inventory tool. Accessed September 1, 2021. https://cmit.cms.gov/CMIT_public/ViewMeasure?MeasureId=5829
9. Quality ID #8 (NQF 0083): Heart Failure (HF): Beta-Blocker Therapy for Left Ventricular Systolic Dysfunction (LVSD). :13.
10. Comprehensive Diabetes Care. NCQA. Accessed March 20, 2021. https://www.ncqa.org/hedis/measures/comprehensive-diabetes-care/
11. US Preventive Services Task Force, Davidson KW, Barry MJ, et al. Screening for Colorectal Cancer: US Preventive Services Task Force Recommendation Statement. *JAMA*. 2021;325(19):1965. doi:10.1001/jama.2021.6238
12. Quality ID #110 (NQF 0041): Preventive Care and Screening: Influenza Immunization. Published online 2019:7.
13. Quality ID #111: Pneumococcal Vaccination Status for Older Adults. Published online 2019:6.
14. Quality ID #134 (NQF 0418): Preventive Care and Screening: Screening for Depression and Follow-Up. :10.
15. Avoidance of Antibiotic Treatment in Adults With Acute Bronchitis. NCQA. Accessed September 1, 2021. https://www.ncqa.org/hedis/measures/avoidance-of-antibiotic-treatment-in-adults-with-acute-bronchitis/
